# Supplementary material for: FERN – a Java framework for stochastic simulation and evaluation of reaction networks
Source: BMC Bioinformatics. 2008 Aug 29;9:356. doi: 10.1186/1471-2105-9-356 (PMC2553347; doi:10.1186/1471-2105-9-356)
Supplement: Additional file 1 — FERN distribution, Version 1.3. This archive contains the FERN source code and binaries as well as documentation and example models in FernML and SBML. [file 1471-2105-9-356-S1.zip › fern/doc/javadoc/index-files/index-9.html]

I-Index


---


|  |  |  |
| --- | --- | --- |
| **Overview** \| Package \| Class \| Use \| **Tree** \| **Deprecated** \| **Index** \| **Help** | |  |
| **PREV LETTER**   **NEXT LETTER** | **FRAMES**    **NO FRAMES**     **All Classes** |  |


A B C D E F G H I J L M N O P R S T U V 

---


## **I**

**img** - Variable in class fern.tools.gnuplot.GnuPlot: **Index** - Variable in class fern.network.sbml.MathTree.VarLeaf: **IndexedPriorityQueue** - Class in fern.simulation.algorithm: Implements an indexed priority queue. **IndexedPriorityQueue(double[])** - Constructor for class fern.simulation.algorithm.IndexedPriorityQueue: Creates a `IndexedPriorityQueue` from the given taus **indexToSpeciesId** - Variable in class fern.network.AbstractNetworkImpl: Stores a mapping from species indices to their names. **inferStringFromSBMLConstant(String, Object)** - Static method in class fern.network.sbml.MathTree: Uses reflection to get a string representation of a libsml constant. **initialAmount** - Variable in class fern.cytoscape.NetworkChecker: **initialize(Network)** - Method in interface fern.analysis.NetworkSearchAction: Gets called before anything is inserted into the search structure. **initialize()** - Method in class fern.simulation.algorithm.GibsonBruckSimulator: **initialize()** - Method in class fern.simulation.algorithm.GillespieEnhanced: **initialize()** - Method in class fern.simulation.algorithm.HybridMaximalTimeStep: **initialize()** - Method in class fern.simulation.Simulator: Initializes the algorithm: set t=0reset the `AmountManager`recalculate the propensities Gets called at the very beginning of `start` **InstantOutputObserver** - Class in fern.simulation.observer: Simply prints out every event that the simulator reports to the observer. **InstantOutputObserver(Simulator, PrintWriter)** - Constructor for class fern.simulation.observer.InstantOutputObserver: Creates the observer for a given simulator and a given `PrintWriter` **interpolateLinear(double, double[], double[])** - Static method in class fern.tools.NumberTools: Performs a linear interpolation of the values. **interpolateLinear(double, double, double, double, double)** - Static method in class fern.tools.NumberTools: Interpolates a value **IntervalObserver** - Class in fern.simulation.observer: Base class for observing certain aspects repeatedly after a given interval. **IntervalObserver(Simulator, double, String[])** - Constructor for class fern.simulation.observer.IntervalObserver: Create the observer for a given simulator, a given interval, given indices with names. **IntQueue** - Class in fern.analysis: IntQueue is an search structure for `AnalysisBase` representing an fifo queue for a breadth first search. **IntQueue()** - Constructor for class fern.analysis.IntQueue: **IntQueue(int)** - Constructor for class fern.analysis.IntQueue: **IntSearchStructure** - Interface in fern.analysis: Implementing class can be used as search structure for searches in `AnalysisBase`. **IntStack** - Class in fern.analysis: IntStack is an search structure for `AnalysisBase` representing an lifo stack for a depth first search. **IntStack()** - Constructor for class fern.analysis.IntStack: **IntStack(int)** - Constructor for class fern.analysis.IntStack: **inverse(String[])** - Static method in class fern.tools.NumberTools: Inverts the given array in place and returns it. **IrreversibleIsomerization** - Class in fern.example: Uses the Irreversible-isomerization model to show effects of different choices for epsilon. **IrreversibleIsomerization()** - Constructor for class fern.example.IrreversibleIsomerization: **isAccessible()** - Method in class fern.tools.gnuplot.GnuPlot: Returns whether or not gnuplot is accessible **isAutocatalyticReaction(int)** - Method in class fern.analysis.AutocatalyticNetworkDetection: Returns true if the given reaction is autocatalytic. **isAutocatalyticSpecies(int)** - Method in class fern.analysis.AutocatalyticNetworkDetection: Returns true if the given species is autocatalytic. **isCountGenerations()** - Method in class fern.tools.Stochastics: Gets whether or not to count the number of random number generations **isEfficientlyAdaptSum()** - Method in class fern.simulation.algorithm.GillespieEnhanced: **isEmpty()** - Method in interface fern.analysis.IntSearchStructure: **isInterpolateTheta()** - Method in class fern.simulation.Simulator: Gets whether the amount values are returned interpolated for theta events. **isPlotQuality()** - Method in class fern.simulation.observer.IntervalObserver: Gets whether or not to plot quality data. **isReactionNode(Node)** - Method in interface fern.cytoscape.NetworkChecker.NodeClassifier: **isReactionNode(Node)** - Method in class fern.cytoscape.NetworkChecker.NodeClassifierByAnnotation: **isReactionToProductEdge(Edge)** - Method in interface fern.cytoscape.NetworkChecker.EdgeClassifier: **isReactionToProductEdge(Edge)** - Method in class fern.cytoscape.NetworkChecker.EdgeClassifierByDirection: **isReactionToProductEdge(Edge)** - Method in class fern.cytoscape.NetworkChecker.EdgeClassifierByIdentifier: **isReactionToReactantEdge(Edge)** - Method in interface fern.cytoscape.NetworkChecker.EdgeClassifier: **isReactionToReactantEdge(Edge)** - Method in class fern.cytoscape.NetworkChecker.EdgeClassifierByDirection: **isReactionToReactantEdge(Edge)** - Method in class fern.cytoscape.NetworkChecker.EdgeClassifierByIdentifier: **isRealTime()** - Method in class fern.cytoscape.ui.OverviewPane: **isSpeciesNode(Node)** - Method in interface fern.cytoscape.NetworkChecker.NodeClassifier: **isSpeciesNode(Node)** - Method in class fern.cytoscape.NetworkChecker.NodeClassifierByAnnotation: **isThetaMethod()** - Method in class fern.simulation.observer.IntervalObserver: Using theta method means that the time intervals are registered at the simulator and the simulator invokes at this moment the method theta. **isUsable()** - Method in interface fern.cytoscape.NetworkChecker.EdgeClassifier: **isUsable()** - Method in class fern.cytoscape.NetworkChecker.EdgeClassifierByDirection: **isUsable()** - Method in class fern.cytoscape.NetworkChecker.EdgeClassifierByIdentifier: **isUsable()** - Method in interface fern.cytoscape.NetworkChecker.NodeClassifier: **isUsable()** - Method in class fern.cytoscape.NetworkChecker.NodeClassifierByAnnotation: **isUsable()** - Method in class fern.cytoscape.NetworkChecker.NodeParameter: **isValid()** - Method in class fern.cytoscape.NetworkChecker: **isVerbose()** - Method in class fern.simulation.algorithm.AbstractBaseTauLeaping: Gets if each step is to print to stdout. **isVisualize()** - Method in class fern.cytoscape.ui.OverviewPane: **iterator()** - Method in class fern.tools.gnuplot.ArrayMatrixAxes: Yields the tab separated columns row by row. **iterator()** - Method in class fern.tools.gnuplot.CollectionAxes: Yields the tab separated columns row by row. **iterator()** - Method in class fern.tools.gnuplot.TransposedArrayMatrixAxes: Yields the tab separated columns row by row.

---


|  |  |  |
| --- | --- | --- |
| **Overview** \| Package \| Class \| Use \| **Tree** \| **Deprecated** \| **Index** \| **Help** | |  |
| **PREV LETTER**   **NEXT LETTER** | **FRAMES**    **NO FRAMES**     **All Classes** |  |


A B C D E F G H I J L M N O P R S T U V 

---
